# Metagenomic Analysis of *Togavirida*e in Mosquito Viromes Isolated From Yunnan Province in China Reveals Genes from Chikungunya and Ross River Viruses

**DOI:** 10.3389/fcimb.2022.849662

**Published:** 2022-02-11

**Authors:** Guanrong Feng, Jinyong Zhang, Ying Zhang, Chenghui Li, Duo Zhang, Yiquan Li, Hongning Zhou, Nan Li, Pengpeng Xiao

**Affiliations:** ^1^ Wenzhou Key Laboratory for Virology and Immunology, Institute of Virology, Wenzhou University, Wenzhou, China; ^2^ Institute of Military Veterinary Medicine, Academy of Military Medical Sciences, Changchun, China; ^3^ College of Veterinary Medicine, College of Animal Science, Jilin University, Changchun, China; ^4^ College of Agriculture, Yanbian University, Yanji, China; ^5^ Academician Workstation of Jilin Province, Changchun University of Chinese Medicine, Changchun, China; ^6^ Yunnan Institute of Parasitic Diseases, Puer, China

**Keywords:** metagenomic analysis, mosquito, virome, virus detection, phylogenetic analysis

## Abstract

We collected 5,500 mosquitoes belonging to six species in three locations in China. Their viromes were tested using metagenomic sequencing and bioinformatic analysis. The affluent viral sequences that were detected and annotated belong to 22 viral taxonomic families. Then, PCR was performed to confirm the results, followed by phylogenetic analysis. Herein, part of mosquito virome was identified, including chikungunya virus (CHIKV), Getah virus (GETV), and Ross river virus (RRV). After metagenomic analysis, seven CHIKV sequences were verified by PCR amplification, among which CHIKV-China/YN2018-1 had the highest homology with the CHIKV isolated in Senegal, 1983, with a nucleotide (nt) identity of at least 81%, belonging to genotype West Africa viral genes. Five GETV sequences were identified, which had a high homology with the GETV sequences isolated from *Equus caballus* in Japan, 1978, with a (nt) identity of at least 97%. The newly isolated virus CHIKV-China/YN2018-1 became more infectious after passage of the BHK-21 cell line to the Vero cell line. The newly identified RRV gene had the highest homology with the 2006 RRV isolate from Australia, with a (nt) identity of at least 94%. In addition, numerous known and unknown viruses have also been detected in mosquitoes from Yunnan province, China, and propagation tests will be carried out.

## 1 Introduction

Mosquitoes are important biological vectors of many infectious viruses, including Getah virus (GETV), Ross River virus (RRV), chikungunya virus (CHIKV), Zika virus (ZIKV), and dengue virus (DENV), which were a menace to global public health and security (Sinclair and Asgari, 2020; Nowee et al., 2021; Pyke et al., 2021). Mosquitoes were considered as an intermediate host of numerous viruses that infect humans, animals, insects, plants, and other species (de Boer et al., 2021). Once mosquitoes bite the host with viremia, they will be infected, and then viruses can replicate and reproduce in the mosquitoes, followed by spread to more victims during the bite and blood-sucking process (Garcia et al., 2018; Martínez et al., 2020). Multiple mosquito-borne viruses exist in Yunnan Province, China (Yu et al., 2021); hence, area surveillance is particularly important. Reverse transcription polymerase chain reaction (RT-PCR) and nested PCR methods were generally employed for detecting mosquito-borne viruses (Cabral-Castro et al., 2016; Morales et al., 2017), while these conventional methods are time-consuming and labor-intensive in the detection of low-level mosquito viromes, compared to Illumina sequencing (He et al., 2013; Martínez-Puchol et al., 2020). Consequently, metagenomic analysis may have important value in avoiding missing detection of highly infectious and pathogenic viruses in different mosquito species, as well as unknown viruses.

The aim of this study was to establish an efficient monitoring method for mosquito-borne viral distribution in Yunnan Province, China, and provide valuable information in the field of viral isolation, prevention, and control. Through metagenomic sequencing and PCR, the diversity and abundant viromes of mosquito-borne viruses in Yunnan province were obtained. The existence of RRV and novel GETV were verified, and CHIKV was isolated. Our initial study on mosquito-borne virus metagenomics provides important technical support for the distribution, diversity, and monitoring of mosquito-borne viruses in China and other countries.

## 2 Materials and Methods

### 2.1 Mosquito Collection

In September and October 2018, 5,500 female mosquitoes, including live and newly dead mosquitoes, were gathered in Yunnan Province, China. The obtained mosquitoes covered *Armigeres obturbans* (*Ar. obturbans*), *Anopheles sinensis* (*An. sinensis*), *Culex quinquefasciatus* (*C. quinquefasciatus*), *Culex tritaeniorhynchus* (*C. tritaeniorhynchus*), *Aedes albopictus* (*Ae. albopictus*), and *Aedes aegypti* (*Ae. aegypti*). After identifying the mosquito species, we put every 100 mosquitoes of the same species in a cryopreservation tube (**Table S1**). Then, based on the collection location, we grouped the mosquito samples (**Table 1**) with storage at −80°C for later use. This study was approved by the Ethics Committee and the Research Ethics Committee of Wenzhou University. All studies relating to active viruses were performed in bio-safety level-3 laboratory.

**Table 1:** Mosquito samples employed in metagenomic analysis and data from Illumina sequencing.

Sample	Species	Number	Location	Total Reads Number	Average (nt)	Viral Reads	Viral Contigs
Sample I	Mosquito Community^#^	3,000	Zhaotong city*	8,422,902	180.62	1,498,434	5,734
Sample II	Mosquito Community^#^	1,400	Puer city*	8,292,559	192.24	602,869	3,218
Sample III	Mosquito Community^#^	1,100	Honghe autonomous prefecture*	7,653,923	179.54	1,441,233	5,175
Total/Average		5,500		24,369,384	184.23	3,542,536	14,127

**Figure 1:**
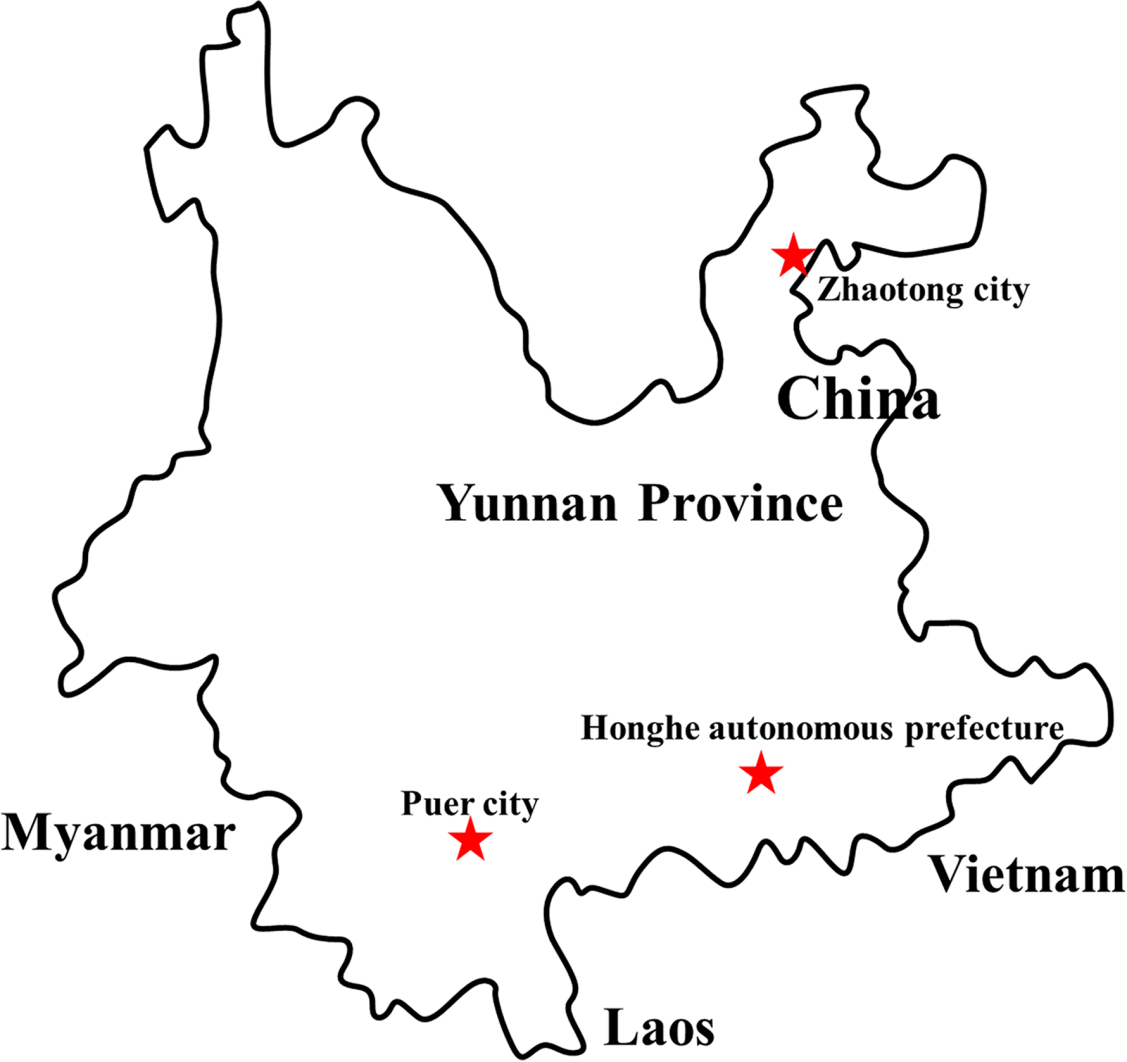
Distribution of sample collection sites in Yunnan province, China, 2018. The sample collection sites were labeled with a red star.

*Collection sites (**Figure 1**) were in Yunnan province including Zhaotong city (N 29° 32’, E 103° 70’), Puer city (N 22° 79′, E 101° 00′), and Honghe autonomous prefecture (N 23° 25′, E 102° 42′). ^#^Mosquito species consisted of C. tritaeniorhynchus, Ar. obturbans, Ae. albopictus, An. sinensis, Ae. aegypti, and C. quinquefasciatus.

### 2.2 Metagenomic Sample Preparation and Metaviral Sequencing

The methods used for metagenomic sample preparation and metaviral sequencing were reference to methods of our previous study (Xiao et al., 2018). The barcode DNA used in metagenomic analysis is shown in **Table 2**.

**Table 2:** Barcode DNA employed in metagenomic analysis (He et al., 2013).

Primer Type	Primer Number	Primers (5’–3’)
Anchored Random Primers	RT1	GCCGGAGCTCTGCAGATATCNNNNNN
	RT 2	GTATCGCTGGACACTGGACCNNNNNN
	RT 3	ATCGTCGTCGTAGGCTGCTCNNNNNN
Barcode Primers	Primer1	GCCGGAGCTCTGCAGATATC
	Primer2	GTATCGCTGGACACTGGACC
	Primer3	ATCGTCGTCGTAGGCTGCTC

### 2.3 Alignment Analysis

The sequences were aligned with non-redundant databases and virus reference databases using BLASTx and BLASTn in GenBank. (https://www.ncbi.nlm.nih.gov/genbank/) The E value ≤10 e^-5^ in BLAST hits was considered statistically significant. Sequences of bacterial and eukaryotes were deleted, and virus-like sequences were analyzed.

### 2.4 Virus-Like Sequence Verification

Viral sequences were aligned with the corresponding viruses in GenBank, the matching positions were found, and then specific primers were designed and synthesized (**Table 3**) for identifying the virus-like sequences. The viral nucleic acid was extracted using a viral nucleic acid extraction kit from Bioer Technology (Hangzhou, China), followed by amplification with PCR Master Mix (Tiangen, Beijing, China) and designed primers.

**Table 3:** Primer pairs used in PCR identification.

Primer Name	Primers (5’–3’)	Product (bp)
CHIKV-China/YN2018E1-1/2/3/4-F	TACGATCAGGTAACTGTGAACC	1,317
CHIKV-China/YN2018E1-1/2/3/4-R	GTGCCTGCTAAACGACACGCATAG	
CHIKV-China/YN2018NS3-F	ATATACTCATCTGACACCGGCC	1,068
CHIKV-China/YN2018NS3-R	AATAATGGCGTCGAAGTCCTCG	
GETV-China/YN2018E2-1/2/3-F	AGTGTGACGGAACACTTCAATGT	1,266
GETV-China/YN2018E2-1/2/3-R	GGGATGCGCTCGTCGTGCGCA	
GETV-China/YN2018NS3-1/2-F	GCACCGTCATACAGCGTCCGC	1,572
GETV-China/YN2018NS3-1/2-R	CGCGCCAGCGCTGCCTAGTGA	
RRV-China/YN2018C-1/2-F	ATGAATTACATACCAACGCAGACT	810
RRV-China/YN2018C-1/2-R	CCACTCTTCGGTTCCTTCTG	
CHIKV-F*	TACGATCAGGTAACTGTGAACC	1,317
CHIKV-R*	GTGCCTGCTAAACGACACGCATAG	

*The primers used in CHIKV identification after viral isolation.

### 2.5 Phylogenetic Analysis

The PCR products were sequenced, and the obtained sequences were used to make an alignment with the representative viruses *via* Clustal W version 2.0. The MEGA 7 was used to build phylogenetic trees based on the tree-building model of maximum-likelihood method with 1,000 bootstrap replicates.

### 2.6 Cell Culture

In this study, BHK-21 and Vero cell lines were cultured in DMEM (HyClone, Logan, UT, USA) supplemented with 10% fetal bovine serum (FBS. HyClone, Logan, UT, USA) and 1% penicillin and streptomycin (Pen Strep. HyClone, Logan, UT, USA); cells were maintained in humidified 5% CO_2_ incubator at 37°C.

### 2.7 Virus Isolation

Mosquito samples verified to be positive by PCR were used for virus isolation. In short, the mosquito sample solution was mixed 1:6 with DMEM containing 2% FBS, followed by inoculation on a monolayer of BHK-21 cells and culture together for 5–6 days. We checked the culture daily for virus-induced cytopathic effect (CPE). Cultures without a CPE were blind passaged 3 times.

### 2.8 PCR and Western Blot Verification of the Isolated Virus

After isolation, the obtained virus CHIKV-China/YN2018-1 was first identified by PCR technology. Based on the envelope 1 (E1) protein gene (**Table 3**), specific paired primers were designed and synthesized. In addition, it was further identified by Western blotting. Anti-E1 monoclonal antibody (Abcam, Cambridge, UK) was used as the primary antibody, and an HRP-conjugated goat anti-mouse antibody (ZSGB-Bio, Beijing, China) was used as the second antibody.

### 2.9 Negative Staining Electron Microscope Observation

After verification by PCR and Western blotting, the newly isolated CHIKV was inoculated on a monolayer of BHK-21 cells to produce CPE. Then, the cells were collected and repeatedly frozen and thawed three times. After that, the virus suspension was harvested by centrifuging at 10,000 × *g* for 15 min, followed by centrifugation at 60,000 × *g* for 4 h. We got the virus precipitate by gently removing and discarding the supernatant, and equal proportion mixture of 6.1% (V/V) glutaraldehyde fixative (pH 7.2) and DMEM was used to resuspend the precipitate, and 25 μl was added to the copper grid, followed by the addition of one drop of 3% phosphotungstic acid for negative staining and observation using a microscope.

### 2.10 The Viral Titer Detection of Different Passages

The viral titers of the 5th- and 10th-generation CHIKV were detected in BHK-21 cells. The CHIKV was treated with serial tenfold dilution and inoculated in cells, and incubated for 120 h at 37°C. The CPE of CHIKV was assessed by using eight replicates in each dilution. The TCID_50_ (50% tissue culture infective dose) was acquired based on the Reed-Muench method (Krah, 1991).

### 2.11 Variation Detection of E1 Gene of Different Passages

CPE was induced in BHK-21 cells with the 5th- and 10th-generation CHIKV. Viral RNA was extracted from the supernatant using an RNA viral kit according to the manufacturer’s protocol (Bioer Technology). After passage in the Vero cell line from the BHK-21 cell line of CHIKV (P5 and P10), the purified RNA was used as template for cDNA synthesis using a SuperScript™ III first-strand synthesis system (Invitrogen) following the manufacturer’s instructions. Subsequently, we amplified the E1 genes of passages 5 and 10 of CHIKV and sequenced them. The four E1 genes were aligned with MEGA 7.0.

### 2.12 Statistical Analysis

All data were input in SPSS 17.0 for statistical analysis. *t*-test was applied in the comparison between groups. Each experiment was set in triplicate, and each independent experiment was repeated three times. It was considered statistically significant when the value of *p* < 0.05.

### 2.13 GenBank Accession Numbers

We have deposited the metagenomic sequencing data in the GenBank Sequence Reads Archive. The obtained accession numbers were SRR15058239 to SRR15058241. We deposited the amplified sequences in the GenBank, whose accession numbers were as follows: CHIKV-China/YN2018E1-1/2/3/4 (MZ494489–MZ494492), CHIKV-China/YN2018NS3-1/2/3/4/5 (MZ494493–MZ494497), GETV-China/YN2018E2-1/2/3 (MZ494498–MZ494500), GETV-China/YN2018NS3-1/2 (MZ494501–MZ494502), and RRV-China/YN2018C-1/2 (MZ494503–MZ494504), respectively.

## 3 Results

### 3.1 Mosquito Virome

**Figure 2:**
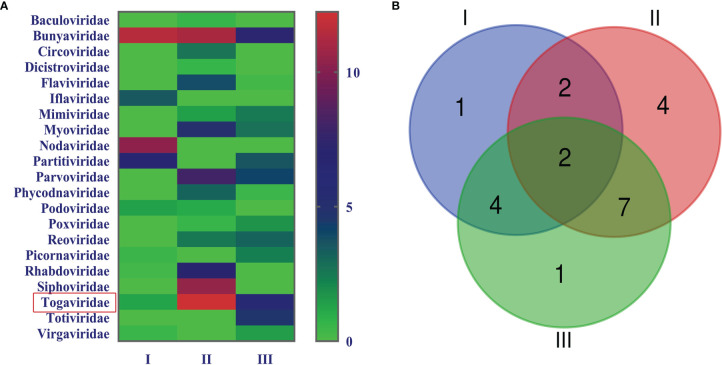
Abundance and relationship of viral families in three samples. The virus sequences were classified based on viral family and displayed relative abundance in the heat map **(A)**. Venn diagram of the number of viral families in the three samples **(B)**.

In order to acquire clean data of mosquito viruses, we eliminated host sequences and barcode DNA. A total of 25,768,166 reads were obtained by Illumina sequencing, and the mean read length was 184.13 nt (**Table 1**). The content of viral sequences in samples I, II, and III accounted for 17.79%, 7.27%, and 18.83%, respectively, of which the relative abundance of viral families is shown (**Figure 2A**), and their intersection of three samples is illustrated (**Figure 2B**). Viral families in samples I, II, and III were 9, 16, and 13 respectively. Interestingly, all three samples contained representative sequences from 2 viral families: *Togaviridae* and *Bunyaviridae.*

### 3.2 PCR Verification of Mosquito Virome

SOAPdenovo was performed to assemble virus sequences into contigs, and 14,127 viral contigs were obtained, of which many virus-like contigs were remarkably similar, such as 63 CHIKV-like contigs with a read coverage of 37× (292–906 nt), 126 GETV-like contigs with a read coverage of 54× (226–872 nt), and 35 RRV-like contigs with a read coverage of 12× (235–843 nt). The CHIKV-like contigs have 92.6%–95.1% nt homology with the known CHIKV sequences. The GETV-like contigs have 83.7%–91.3% nt homology with the known GETV sequences. The RRV-like contigs have 81.6%–90.5% nt homology with the known RRV sequences. Next, we designed and synthesized specific primers to amplify the identified viruses, to further confirm the results of metavirome sequencing (**Table 3**). The results of PCR verification of mosquito virome were as follows.

#### 3.2.1 PCR Verification of CHIKV

**Figure 3:**
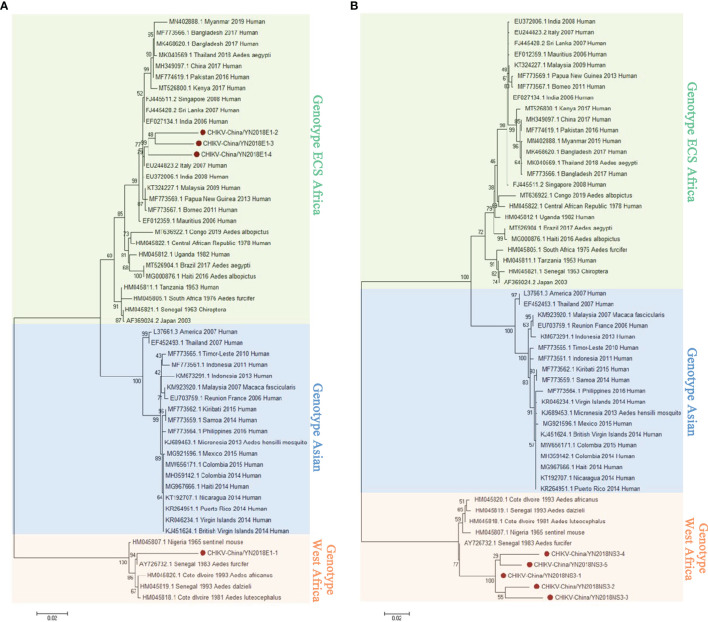
The CHIKV phylogenetic trees. E1 **(A)** and NS3 **(B)** gene of CHIKV used to build Phylogenetic trees. The maximum likelihood method was conducted with MEGA 7.0. The Bootstrap value was calculated through 1,000 repetitions. Use solid red circles to mark the genes identified in this research.

In keeping with the comparison results of viral contigs, the viral PCR amplicons from sample II had a high homology with CHIKV assigning to *Alphavirus* of *Togaviridae*. The results were verified by RT-PCR. Four 1,317-nucleotide-long segments (CHIKV-China/YN2018E1-1/2/3/4) and five 1,068-nucleotide-long segments (CHIKV-China/YN2018NS3-1/2/3/4/5) were amplified, and shared ~81%–95% and ~94%–98% nucleotide (nt) identity and ~83%–88% and ~86%–95% amino acid (aa) with each other, respectively. We aligned these segments using BLASTN against the GenBank, and identified that they shared high identity with the E1 gene and the non-structural protein 3 (NS3) of CHIKV from Senegal isolated in 1983, with at least 81% and 80% nt identity and 83% and 83% aa identity, respectively, suggesting that these viral genes from a CHIKV variant (**Figure 3**). Phylogenetic analysis of the newly identified CHIKV sequences demonstrated that they assigned to genotype ECS and West Africa viral genes.

#### 3.2.2 PCR Verification of GETV

**Figure 4:**
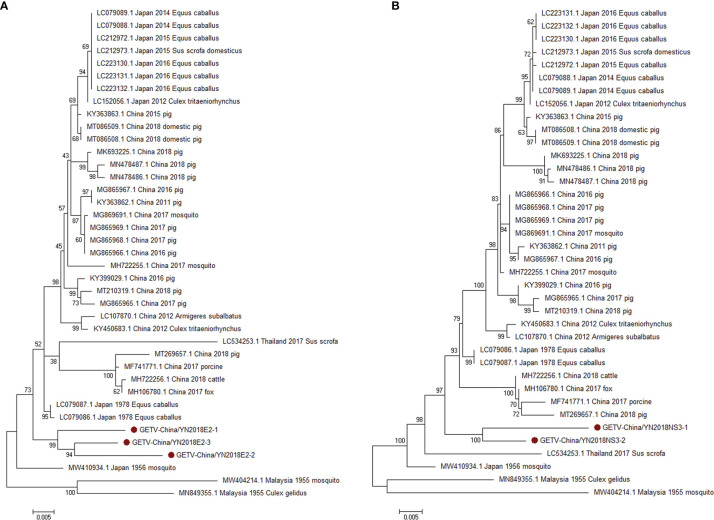
The phylogenetic trees of GETV. E2 **(A)** and NS3 **(B)** gene of GETV used to build Phylogenetic trees. The maximum likelihood method was conducted with MEGA 7.0. The Bootstrap value was calculated through 1,000 repetitions. Use solid red circles to mark the genes identified in this research.

According to the viral contigs alignment results, PCR amplicons of GETV-like contigs in sample III had a high homology with GETV assigning to *Alphavirus* of *Togaviridae*. Three 1,266-nucleotide-long segments (GETV-China/YN2018E2-1/2/3) and two 1,572-nucleotide-long segments (GETV-China/YN2018NS3-1/2) were amplified, and shared ~96%–97% and ~97% nt identity and ~91%–93% and ~93% aa identity with each other, respectively. We aligned these segments using BLASTN against the GenBank, and identified that they shared high identity with the envelope 2 (E2) gene and the NS3 gene of GETV sequence from *Equus caballus* isolated in Japan, 1978, with at least 97% and 97% nt identity and 93% and 93% aa identity, respectively, suggesting they were from a GETV variant (**Figure 4**).

#### 3.2.3 PCR Verification of RRV

**Figure 5:**
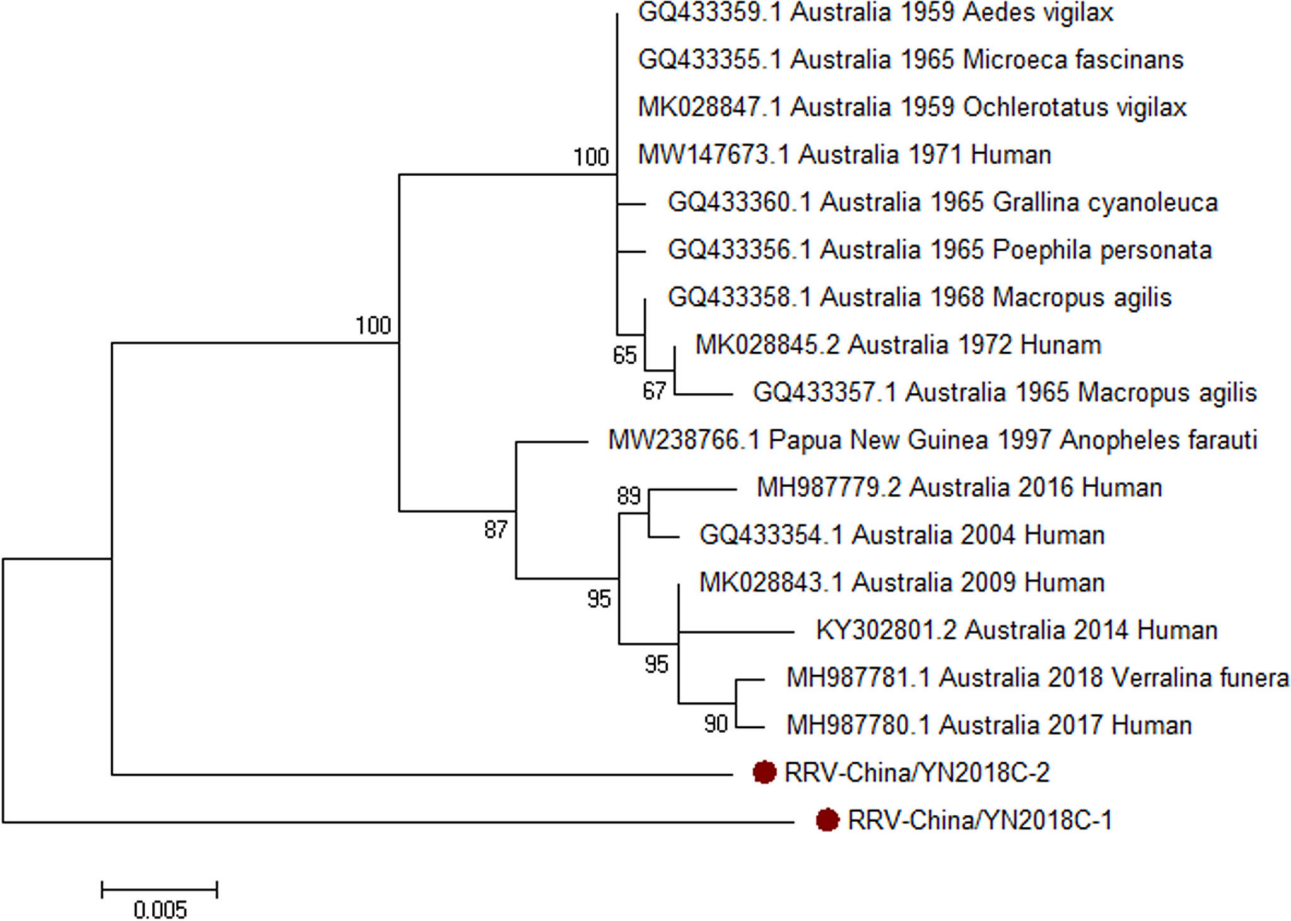
The phylogenetic trees of RRV. C gene of RRV used to build phylogenetic trees. The maximum likelihood method was conducted with MEGA 7.0. The Bootstrap value was calculated through 1,000 repetitions. Use solid red circles to mark the genes identified in this research.

Armed with the results of viral contigs alignment, PCR amplicons of RRV-like contigs in sample II had a high homology with RRV assigning to *Alphavirus* of *Togaviridae*. Two 810-nucleotide-long segments (RRV-China/YN2018C-1/2) were amplified, and shared ~94% nt identity and ~87% aa identity with each other, respectively. We aligned these segments using BLASTN against the GenBank, and identified that they shared high identity with the capsid protein (C) gene of RRV, with at least 94% nt identity and 89% aa identity, respectively, with the RRV sequence from human isolated in Australia, 2006 (**Figure 5**).

### 3.3 Viral Isolation Identification of CHIKV

**Figure 6:**
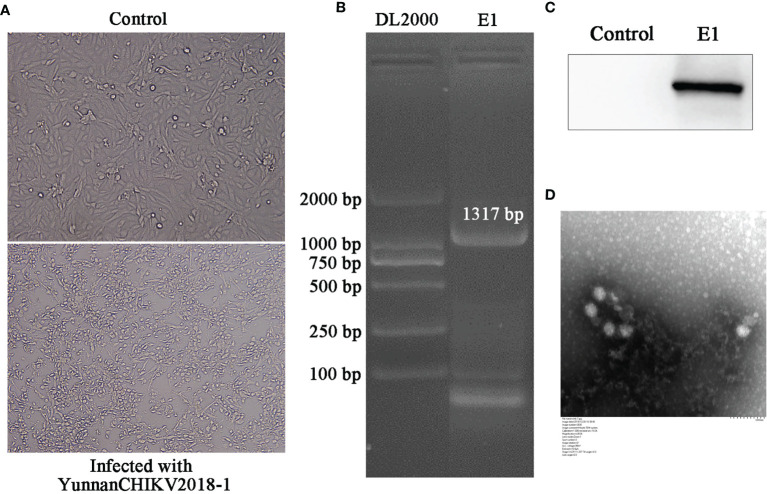
Identification of *CHIKV*-China/YN2018-1 isolation in Honghe autonomous prefecture of Yunnan province by PCR, Western blot, and negative-stain electron microscopy. CPE observation of *CHIKV*-China/YN2018-1 after BHK-21 cell infection **(A)**. PCR identification of *CHIKV*-China/YN2018-1 after BHK-21 cell infection **(B)**. Western blot identification of *CHIKV*-China/YN2018-1 isolation strain was assessed with an anti-E monoclonal antibody (Abcam, Cambridge, UK) and an HRP-conjugated goat anti-mouse antibody (ZSGB-Bio, Beijing, China) **(C)**. Negative-stain electron microscopy of *CHIKV*-China/YN2018-1 particles **(D)**.

Considering the successful amplification of CHIKV in sample II, we tried to validate all mosquito samples from sample II and then detected and isolated CHIKV from *Aedes albopictus* samples. The obtained CHIKV strain was first detected by PCR experiment. Then, Western blot was performed to further detect the levels of intracellular virus (E1 protein). As predicted, E1 gene/protein was positively expressed in CHIKV-infected cells rather than mock-infected cells. In order to further verify the isolation of CHIKV, we observed the virus particles through negative-stain electron microscopy, which were shown rounded with approximately 70 nm diameter and tiny protrusions on the surface, which presented CHIKV-like particles (**Figure 6**).

### 3.4 The Viral Titer of Different Passage of CHIKV-China/YN2018-1

After inoculation in BHK-21 cells and calculation, the viral titers of CHIKV-China/YN2018-1 of the 5th and 10th generation were 2.65 × 10^4^ TCID_50_/0.1 ml and 5.14 × 10^4^ TCID_50_/0.1 ml, which suggested that multiple passages might lead to the change of viral titers.

### 3.5 The Variability of CHIKV-China/YN2018-1 E Gene of Different Passages

After sequencing, MegAlign was utilized to align the CHIKV-China/YN2018-1 E genes of P5 and P10. Nucleotide identity analysis showed that E1 gene of P5 shared 99.2% nucleotide identity with that of P10. Then, follow-up infection was performed on Vero cell lines, and the 5th and 10th generations of CHIKV-China/YN2018-1 E genes were sequenced. Nucleotide comparison analysis showed that 9 nucleotide sites of P5 and 14 nucleotide sites of P10 of E genes presented polymorphism. Alignment of the amino acid sequence showed that one vital amino acid site (position: 226 aa) of E1 protein in P5 and P10 was mutated, with amino acid A (alanine) changed to V (valine). Moreover, after detecting the viral titers in Vero cells, the P5 and P10 of CHIKV-China/YN2018-1 were 1.67 × 10^5^ TCID_50_/0.1 ml and 2.14 × 10^5^ TCID_50_/0.1 ml, which were markedly elevated (*p* < 0.05) in comparison with detection in BHK-21 cells.

## 4 Discussion

Mosquitoes are the intermediate host of many viruses, playing an important part in the spread of numerous viral infectious diseases (Zhu et al., 2019; Estrada-Franco et al., 2020). Compared with the traditional method, the efficiency of metagenomic sequencing by Illumina sequencing combined with bioinformatics analysis is higher, and in this way, many viruses from different species have been identified in recent years (Zhang et al., 2018; Marzoli et al., 2021; Thannesberger et al., 2021). In addition, the component of mosquito virome and the high abundance of viruses in mosquitoes can be revealed using metagenomic sequencing (Metsky et al., 2019; Ramírez et al., 2020). Moreover, metagenomic sequencing is also of great significance for the discovery and identification of novel viruses (Sadeghi et al., 2018; Santos et al., 2021). Many mosquito species virome studies applying NGS tools identified new viruses and described their diversity, which led to the discovery of insect-specific viruses (Romo et al., 2018; Chenyan et al., 2020). We mainly studied mosquito viromes in different cities, hinting at the prevalence of different viruses in specific cities. In this study, we detected up to 21 viral families in mosquito samples using Illumina sequencing. However, the percentage of virus sequences obtained from the three mosquito samples varies greatly. This may be related to the geographical location of mosquito species (Bang et al., 2021; Khalil et al., 2021). Although the mosquitoes from multiple geographic locations were pooled, some viruses can be discovered in non-*Ae. aegypti/Ae. albopictus* mosquito species (Kamgang et al., 2020; Bennett et al., 2021). Despite that, we obtained novel discoveries about the distribution and evolution of mosquito viromes in Yunnan Province, China.

We verified the results of Illumina sequencing using RT-PCR and PCR. RRV amplification in the mosquito samples showed that RRV was first detected in Yunnan province, China, which suggested that there was a potential epidemic of RRV in this area. Moreover, the successful amplification of the new GETV was of great significance, as it was highly infectious to humans and animals, especially horses. In addition, the detection of unidentified viruses in the Illumina sequencing results might be related to insufficient mosquito sampling and limited collection sites.

We simultaneously amplified CHIKV and RRV in mosquito samples, indicating that the two viruses co-transmitted in Yunnan province, and the viruses may be co-infected. Although the discovery were not enough to prove the co-infection of the viruses, it was still of great value to detect the co-circulation of RRV and CHIKV in the same geographic location. The co-circulation of the two viruses may present new challenges for the prevention and control of CHIKV.

The isolation and identification of CHIKV reflected that the virus was still spreading and circulating in Yunnan province. According to the alignment results of CHIKV-China/YN2018-1 of P5 and P10 against those isolated from infected Vero cells, one amino acid site (position: 226 aa) of E1 proteins in P5 and P10 both mutated, with amino acid A (alanine) changed to V (valine). It has been reported that E1-A226V mutation at the position of 226 in E1 protein can significantly improve the adaptability of CHIKV to *Ae. albopictus* (Agbodzi et al., 2021). Then, we found that the viral titers of CHIKV-China/YN2018-1 of P5 and P10 had a significant increase. The results showed that infectivity of CHIKV-China/YN2018-1 was enhanced when passaged in Vero cells from BHK-21, implying that CHIKV had the potential to spread across the species barrier and increase infectivity, thereby increasing the threat to human health.

It was obvious that our results indicated that the distribution of viruses detected in mosquitoes obtained from Yunnan province depends on the geographical location of mosquitoes. However, our current research explored only a small part of the mosquito viromes in this area; further research is needed in more regions or countries to explore the abundance and diversity of the mosquito viromes. In general, our research has brought useful insights for virus isolation and identification of CHIKV and new GETV, which potentially will be used in the field of viral epidemiology.

## Data Availability Statement

The datasets presented in this study can be found in online repositories. The names of the repository/repositories and accession number(s) can be found in the article/**Supplementary Material**.

## Author Contributions

PX and NL conceived and designed the experiments. GF, JZ, CL, YZ, and DZ performed the experiments. PX and YL analyzed the data. HZ contributed reagents/materials/analysis tools. PX and NL wrote the paper. All authors contributed to the article and approved the submitted version.

## Funding

This work was supported by the National Natural Science Foundation of China [grant number 32002312] and the Wenzhou Basic Scientific Research Project [grant number Y2020103].

## Conflict of Interest

The authors declare that the research was conducted in the absence of any commercial or financial relationships that could be construed as a potential conflict of interest.

## Publisher’s Note

All claims expressed in this article are solely those of the authors and do not necessarily represent those of their affiliated organizations, or those of the publisher, the editors and the reviewers. Any product that may be evaluated in this article, or claim that may be made by its manufacturer, is not guaranteed or endorsed by the publisher.
