# Supplementary material for: Metagenomic Analysis of Togaviridae in Mosquito Viromes Isolated From Yunnan Province in China Reveals Genes from Chikungunya and Ross River Viruses
Source: Front Cell Infect Microbiol. 2022 Feb 11;12:849662. doi: 10.3389/fcimb.2022.849662 (PMC8878809; doi:10.3389/fcimb.2022.849662)
Supplement: Supplementary file 1 [file Table_1.docx]

**Table S1.** **The profile of mosquito samples employed in metagenomic analysis.**

| **Sample** | **Species** | **Number** | **Total Number** | **Location** | | **GPS coordinates** |
| --- | --- | --- | --- | --- | --- | --- |
| Sample I | *Culex tritaeniorhynchus* | 1873 | 3000 | Zhaotong city | | N 29° 32’, E 103° 70’ |
|  | *Armigeres obturbans* | 504 |  |  |  |  |
|  | *Aedes albopictus* | 216 |  |  |  |  |
|  | *Anopheles sinensis* | 289 |  |  |  |  |
|  | *Culex quinquefasciatus* | 118 |  |  |  |  |
| Sample II | *Culex tritaeniorhynchus* | 814 | 1400 | Puer city | | N 22° 79′, E 101° 00′ |
|  | *Aedes albopictus* | 326 |  |  |  |  |
|  | *Aedes aegypti* | 227 |  |  |  |  |
|  | *Culex quinquefasciatus* | 33 |  |  |  |  |
| Sample III | *Culex tritaeniorhynchus* | 551 | 1100 | Honghe autonomous prefecture | | N 23° 25′, E 102° 42′ |
|  | *Armigeres obturbans* | 239 |  |  |  |  |
|  | *Aedes aegypti* | 175 |  |  |  |  |
|  | *Anopheles sinensis* | 135 |  |  |  |  |
| Total |  |  | 5500 | |  |  |
